# Distinguishing early from late mild cognitive impairment: a multi-level analysis of regional morphometry and KLS-derived network topology

**DOI:** 10.3389/fnagi.2026.1730305

**Published:** 2026-03-11

**Authors:** Peng Yan, Xinyu Du, Siyu Yang

**Affiliations:** 1 Department of Neurology, The First Affiliated Hospital with Nanjing Medical University, Nanjing, Jiangsu, China; 2 Department of Neurology, The Second Hospital of Nanjing, Affiliated to Nanjing University of Chinese Medicine, Nanjing, Jiangsu, China

**Keywords:** individual structural covariance network, Kullback-Leibler similarity, machine learning, mild cognitive impairment, structural magnetic resonance imaging

## Abstract

**Introduction:**

Distinguishing between early Mild Cognitive Impairment (EMCI) and late mild cognitive impairment (LMCI) is crucial for clinical trials, but objective biomarkers are lacking. We therefore examined regional morphometry and network topology across cognitively normal (CN), EMCI, and LMCI groups to address this gap. We also evaluated whether combining these features could effectively classify mild cognitive impairment (MCI) subtypes.

**Methods:**

We analyzed T1-weighted magnetic resonance imaging (MRI) data from 208 Alzheimer’s Disease Neuroimaging Initiative (ADNI) participants (67 CN, 83 EMCI, 58 LMCI). We used both voxel- and surface-based morphometry to measure local atrophy and combined this with graph analysis of individual structural covariance networks (SCNs). We also performed correlation and machine learning analyses.

**Results:**

We found that cortical thickness (CT) in EMCI was not significantly different from CN, but it was significantly reduced in the LMCI group. The right hippocampus and the left thalamus, however, showed a significant difference between CN and EMCI. In the Kullback–Leibler (KL) divergence-based similarity (KLS) network analysis, the EMCI group showed a greater randomization when compared to the LMCI group, while LMCI was accompanied by elevated nodal centrality in the left hippocampus and orbital frontal region. Correlation analysis confirmed this was a maladaptive phenomenon, as higher centrality was linked to poorer cognitive performance. Finally, a classifier combining these structural and network features successfully differentiated the MCI subtypes.

**Conclusion:**

Our findings suggest that differences in Gray matter volume (GMV) may be more easily observed in the EMCI group. We identified a corresponding non-linear pattern of network topology, characterized by randomization in the EMCI group than in the LMCI. These multi-faceted biomarkers enabled the accurate machine-learning-based differentiation of MCI subtypes, offering a powerful framework for improving patient stratification in clinical trials.

## 1 Introduction

Alzheimer’s disease (AD) is the most common form of dementia. The number of people with the disease is expected to reach 82 million by 2030, and could even climb to 152 million by 2050 (van der Flier et al., 2024; Zhang and Zhang, 2024). As there are no treatments to reverse AD progression currently, early diagnosis of AD has become a major clinical priority. The clinical syndrome of mild cognitive impairment (MCI) is defined by objective evidence of cognitive decline beyond what is typical for a person’s age and education, in the absence of significant functional impairment in daily living (Ding et al., 2024; Gauthier et al., 2006). MCI is often staged as either early (EMCI) or late (LMCI) to reflect the severity of cognitive and functional decline, with LMCI representing a more advanced state of impairment (Gauthier et al., 2006; Feng et al., 2025). The annual conversion rate from MCI to dementia ranges from 8 to 15%, with the cumulative rate exceeding 80% after 6 years (Blanco et al., 2023). MCI subtypes present varied risks for subsequent AD development (Ward et al., 2021). Differentiating early from late mild cognitive impairment (EMCI vs. LMCI) is a major clinical hurdle, the prediction of MCI relies on many biomarkers such as neuroimaging and fluid-based assays (Mahmud et al., 2018; Mahmud et al., 2021). The low cost, short scan times, and robustness against motion artifacts make structural magnetic resonance imaging (sMRI) a highly suitable tool for assessing brain structure (Yan et al., 2023). In addition to widely-used methods like Voxel- and Surface-Based Morphometry (VBM & SBM), structural covariance networks (SCNs) offer a powerful way to investigate differences in gray matter covariance patterns under various pathological conditions (Lai et al., 2023).

Standard morphometric approaches, such as VBM, can effectively detect localized gray matter atrophy. Yet these techniques have an important blind spot: they overlook the interconnected nature of the brain. Brain regions do not operate in isolation; instead, they are closely linked (Damoiseaux et al., 2006). This reality has motivated researchers to shift toward network-based analyses, which aim to capture the statistical dependencies among different brain areas (Zugman et al., 2021). One promising tool in this regard is the structural covariance network (SCN), which quantifies how morphological patterns in one region relate to those in another. SCNs have shown sensitivity to early AD pathology, such as amyloid-β deposition, and may therefore serve as non-invasive biomarkers for detecting MCI (Tijms et al., 2015; Pereira et al., 2016). Early SCN studies constructed networks at the group level, aggregating data across many individuals to produce a single representative network (Raj et al., 2010; Tijms et al., 2011). Tijms and colleagues, for instance, developed a voxel-based approach grounded in axonal tension theory—the idea that axonal connections shape cortical morphology (Hilgetag and Barbas, 2005). Their method uses fine-grained cubic resolutions (3 × 3 × 3 voxels) to build individual networks. While this preserves local detail, it imposes a rigid spatial grid that may not align well with the natural folding of the cortex (Tijms et al., 2011). More fundamentally, group-level SCN approaches average out individual variability, making it difficult to link network features to specific clinical or cognitive outcomes in single patients (Alexander-Bloch et al., 2013). Kong et al. addressed this limitation by introducing a framework for constructing SCNs at the individual level (Kong et al., 2014). Their approach relies on Kullback-Leibler (KL) divergence to quantify the similarity between probability distributions of gray matter density across brain regions. Since standard KL divergence is asymmetric, they symmetrized it to yield a stable metric known as KL-based similarity (KLS). This method has proven reliable in test-retest studies (Wang et al., 2016) and has been applied to a range of conditions, from migraine (Chen W. et al., 2024), social anxiety disorder (Zhang et al., 2023), and MCI (Xu et al., 2024). Building on this work, Xu et al. (2024) recently showed that individual SCNs constructed using Jensen-Shannon divergence can distinguish MCI patients from controls. An important unanswered question, however, is whether such networks can also differentiate MCI subtypes—specifically, early MCI (EMCI) versus late MCI (LMCI).

Our study extends this line of investigation in several ways. First, rather than relying solely on gray matter volume, we incorporated multiple surface-based metrics: cortical thickness (CT), sulcal depth (SD), fractal dimension (FD), and the gyrification index (GI). Each of these features has been independently associated with AD pathology and cognitive decline (Pérez-Millan et al., 2023; Lau et al., 2023; Gan et al., 2024; Lin et al., 2025; Sim et al., 2025), and combining them may reveal subtle degenerative patterns that volumetric measures alone would miss. Second, we adopted the KLS similarity measure developed by Kong et al. (2014) for two main reasons: it has undergone more extensive validation than Jensen-Shannon divergence, and it has shown robust performance across multiple neurological and psychiatric conditions, such as Parkinson’s disease (Yan et al., 2023; Suo et al., 2021), migraine (Chen W. et al., 2024), and social anxiety disorder (Zhang et al., 2023). Third, we imposed strict diagnostic criteria by requiring amyloid positivity in all MCI participants. This aligns with current NIA-AA guidelines and ensures that our sample consists of patients on the AD trajectory, rather than a mix of etiologies. Finally, we adopted a multi-level analytical strategy. We first mapped regional atrophy patterns using VBM and SBM to establish an anatomical foundation, then extended this to the network level by constructing individual SCNs to reveal topological disruptions. Ultimately, we integrated these multi-level features to assess their combined utility in classifying MCI subtypes and their clinical relevance.

## 2 Materials and methods

### 2.1 Sample characteristics

All participants were selected from the Alzheimer’s Disease Neuroimaging Initiative (ADNI) database. We excluded individuals with significant cerebrovascular disease (Hachinski Ischemic Score > 4) or clinically significant depression (Geriatric Depression Scale > 6), and then we selected participants aged 55–90 who had a high-quality T1-weighted MRI and available APOE genotype. To ensure data consistency, we only used scans that met specific acquisition parameters (see MRI Acquisition for details). Following established guidelines, we defined amyloid-positivity (A+) as a Cerebrospinal Fluid (CSF) Aβ1-42 concentration below 192 pg/mL (Shaw et al., 2011). For PET scans (using either FBP or FBB tracers), we classified individuals as amyloid-positive (A+) if their summary SUVR was above the established thresholds (1.11 for FBP; 1.08 for FBB) (Landau et al., 2013; Ossenkoppele et al., 2018). If the PET and CSF results were in conflict, the CSF finding was considered definitive. Participants with a negative CSF result were defined as amyloid-negative (A-).

We classified participants into three groups, the Cognitively Normal (CN) group consisted of amyloid-negative (A-) individuals. Both EMCI and LMCI participants were required to be amyloid-positive (A+). The final distinction between these groups was based on their Logical Memory II scores, which we stratified by years of education (16+, 8–15, and 0–7). Specifically, CN participants scored above the impairment thresholds ( ≥ 9, ≥ 5, ≥ 3); EMCI participants scored within a defined range of mild impairment (9–11, 5–9, 3–6); and LMCI participants scored below this range ( ≤ 8, ≤ 4, ≤ 2). This classification process resulted in a final sample of 208 individuals: 67 CN, 83 EMCI, and 58 LMCI.

### 2.2 MRI data acquisition and processing

We analyzed baseline T1-weighted MPRAGE scans from ADNI-GO and ADNI-2. To ensure data homogeneity, we exclusively selected scans acquired on 3.0T SIEMENS scanners. All selected scans shared consistent acquisition parameters, including a slice thickness of 1.2 mm and 176 axial slices (Jack et al., 2008). The CAT12 Toolbox^1^ in MATLAB (R2018a) facilitated all subsequent preprocessing and morphometric analyses. We used its adaptive maximum a posteriori estimation and local intensity adaptation algorithms for initial segmentation of T1-weighted scans into gray matter, white matter, and cerebrospinal fluid (Dahnke et al., 2012). The GM segmentations were aligned, resampled to a 1.5 mm isotropic voxel resolution, and transformed into Montreal Neurological Institute (MNI) space via the Diffeomorphic Anatomical Registration Through Exponential Lie (DARTEL) algorithm. We employed 8-mm full-width at half-maximum (FWHM) Gaussian smoothing to the normalized gray matter maps. Finally, stringent quality control was performed through visual inspection of all segmentations and reconstructions, and by using the automated quality assurance (QA) report generated by CAT12. Only subjects with an image-quality rating above “C” were included in the statistical analyses.

### 2.3 Network construction

We defined the structural relationship between any two brain regions by quantifying the similarity of their underlying morphological probability distributions, allowing us to construct SCNs for each individual subject (Zhang et al., 2023). We constructed GMV-based SCNs by parcellating whole-brain gray matter into 116 ROIs according to the Automated Anatomical Labeling (AAL116) atlas (Tzourio-Mazoyer et al., 2002); these ROIs served as network nodes. For SBM-based networks—encompassing CT, SD, FD, and GI—we applied the Desikan-Killiany (DK) atlas (Potvin et al., 2017) to define 68 cortical ROIs as nodes. Within each ROI, we estimated the probability density function (PDF) of the local morphometric values (voxel-wise for GMV; vertex-wise for SBM metrics). PDF estimation was performed using a publicly available MATLAB toolbox^2^ for kernel density estimation. This toolbox uses a Gaussian kernel and selects the bandwidth automatically based on the data (Botev et al., 2010). Specifically, for each ROI, we extracted all voxel- or vertex-wise morphometric values as sample data and applied kernel density estimation (KDE) to obtain a non-parametric estimate of their underlying probability distribution. We used a Gaussian kernel for the KDE, and the bandwidth was determined automatically by an adaptive algorithm that balances smoothness against accuracy. All PDFs were then sampled on a fixed grid, which we defined based on the minimum and maximum values across all ROIs for each subject. This ensured that PDFs from different regions could be directly compared. Because zero-valued probabilities can create numerical problems when calculating Kullback-Leibler divergence, we added a small positive constant to all density estimates. The KL divergence between each pair of ROI PDFs was then computed to quantify distributional differences; these values formed the network edges:


$$D_{\text{KL}}\left(P||Q\right)=\overset{\text{n}}{\underset{\text{i}=1}{\sum }}P\left(i\right)\log{\frac{P\,\left(i\right)}{Q\,\left(i\right)}}_{,}$$

Given the inherent asymmetry of standard KL divergence (i.e., *D*_KL_(*P*||*Q*) typically does not equal *D*_KL_(*Q*||*P*)), we employed a symmetric KL divergence, *D*_KL_(*P*,*Q*) (Kong et al., 2014; Wang et al., 2016; Homan et al., 2019), to assess the bidirectional similarity between two PDFs (P and Q). This approach ensures a direction-independent measure of inter-regional similarity, and its calculation is as follows:


$$D_{\text{KL}}\,\left(P,Q\right)=\overset{\text{n}}{\underset{\text{i}=1}{\sum }}\left(P\,\left(i\right)\,\text{log}\,\frac{P\,\left(i\right)}{Q\,\left(i\right)}+Q\,\left(i\right)\,\text{log}\,\frac{Q\,\left(i\right)}{P\,\left(i\right)}\right)$$

Subsequently, the calculated symmetric KL divergence values were transformed into a standardized similarity measure, known as Kullback-Leibler Similarity (KLS). KLS values range from 0 (indicating completely dissimilar distributions) to 1 (indicating identical distributions). This transformation was achieved using the following exponential function (Kong et al., 2014; Wang et al., 2016):


$$\text{KLS}\,\left(P,Q\right)=e^{-D_{\text{KL}}\,\left(P,Q\right)}$$

This procedure was performed for all possible pairwise combinations of ROIs within each atlas. For the VBM data, we generated a 116 × 116 KLS similarity matrix derived from the AAL atlas for each subject. Similarly, for each of the four SBM metrics, a 68 × 68 KLS similarity matrix was constructed based on the DK atlas. The diagonal elements of these matrices were set to 1. Each resulting KLS similarity matrix was defined as an individual’s structural covariance network (SCN) for the corresponding morphological feature.

### 2.4 Network analyses

We performed graph theoretical analyses using the GRETNA toolbox^3^. First, we converted the continuous KLS similarity matrices into binary networks via a sparsity-based thresholding procedure. This ensured all networks had the same number of edges at a given sparsity (ratio of actual to possible edges). Specifically, We applied a range of sparsity thresholds (from 0.05 to 0.30 in 0.01 increments) to create the 116 × 116 GMV-based SCNs. For the smaller 68 × 68 surface-based networks, we adjusted the starting threshold to 0.07 to ensure the graphs remained connected. This process yielded binary networks where an edge value of 1 marked a significant morphological covariation between regions, and 0 marked its absence.

We characterized each subject’s network across sparsity by combining global and node-wise measures. These included small-worldness, global efficiency, local efficiency, characteristic path length, and the clustering coefficient. To normalize the characteristic path length and clustering coefficient, we also generated 1000 matched random networks. In parallel, we calculated several nodal metrics, including nodal degree, nodal efficiency, and nodal betweenness centrality. Finally, we calculated the area under the curve (AUC) for each metric across the entire sparsity range to avoid potential biases from any single threshold (Panhwar et al., 2022).

### 2.5 Statistical analyses

We conducted all statistical analyses using R software (version 4.2.2).

#### 2.5.1 Characterization of demographic and clinical data

To characterize our sample, we evaluated group differences in demographic and clinical attributes. For continuous variables, we employed one-way analysis of variance (ANOVA), whereas chi-square (χ^2^) tests were utilized for categorical factors. A *P*-value below 0.05 was considered statistically significant for these comparisons.

#### 2.5.2 Statistical analysis of neuroimaging data

To investigate group differences in brain structure and network organization, a series of one-way analyses of covariance (ANCOVA) were conducted. Unless otherwise specified, all ANCOVA models controlled for the potential confounding effects of age, sex, and education years. APOE ε4 carrier status, defined as a binary variable (0 for non-carriers, 1 for carriers of at least one ε4 allele), was also included as a covariate. For analyses involving VBM-derived metrics (i.e., GMV-based networks), total intracranial volume (TIV) was included as an additional covariate to account for variations in head size. For VBM, whole-brain voxel-wise ANCOVA was performed on smoothed, modulated gray matter maps with a voxel-level threshold of *P* < 0.001 (uncorrected) and a cluster-level threshold of *P*_*FWE*_ < 0.05. For SBM, vertex-wise ANCOVA was conducted on cortical surface maps (e.g., cortical thickness) using the same cluster-level correction. Mean values were extracted from significant clusters for subsequent *post-hoc* comparisons.

#### 2.5.3 Analysis of network topological properties

Group differences in the Area Under the Curve (AUC) of both global and nodal network metrics were also assessed using ANCOVA. For the small number of global metrics, statistical significance was set at *P* < 0.05. For the multiple nodal metrics, FDR correction (*P* < 0.05) was applied across all nodes for each metric separately.

We tested group differences in morphological connectivity for each edge derived from the individual Kullback–Leibler–based similarity matrices using analysis of covariance. We controlled multiple comparisons with the false discovery rate procedure (*P* < 0.05). For edges that survived correction, we reported effect sizes: partial eta squared for the main group effect and Cohen’s d for each pairwise contrast. Because VBM showed atrophy in the bilateral hippocampi and the left thalamus, we then extracted all significant connections involving these regions for a focused, region-of-interest review. Next, we examined clinical relevance. We correlated the significant VBM, SBM, and node-level network metrics with the primary cognitive scores. For connections, we kept the scope narrow and tested only links between our regions of interest and the rest of the brain. We corrected all correlation *p*-values using the false discovery rate.

#### 2.5.4 Correlation analyses

We ran a series of Pearson’s correlation analyses between our imaging metrics and the primary cognitive scores and key AD biomarkers. First, we correlated all significant VBM, SBM, and nodal metrics with cognitive performance as well as with cerebrospinal fluid (CSF) biomarkers [amyloid-beta 42 (Aβ42), phosphorylated tau (p-tau), total tau (t-tau)] and amyloid PET standardized uptake value ratio (SUVR). For the network connections, we adopted a more focused approach to avoid the statistical challenges of a whole-brain analysis. We exclusively analyzed connections between our predefined regions of interest (the left and right hippocampus and the left thalamus) and the rest of the brain. From this set, we selected only those connections that showed a significant correlation with cognition to use as features for our subsequent machine learning model. We then used these selected features to train a Logistic Regression (LR) model to classify the participant groups.

### 2.6 Machine learning analyses

To further validate the discriminative power of the identified imaging features and conduct a preliminary exploration of their potential diagnostic value, we performed pairwise classification analyses using a Logistic Regression (LR) model. In brief, we vectorized a focused set of 13 local imaging features (selected based on their significant correlation with ADAS-Cog13 scores) as input for subsequent analyses; in each iteration of the outer cross-validation, per-feature covariate residualization was performed solely on the training set data, followed by min-max normalization on the residualized feature matrix. Crucially, within each outer training fold, feature selection was performed via L1-regularized Logistic Regression (LASSO), where only features with non-zero coefficients were retained for the final model. Machine learning employed a nested cross-validation (CV) scheme, with an outer loop of 10-fold stratified CV and an inner loop of 5-fold stratified CV to optimize the LR model’s hyperparameters. With hyperparameter tuning finalized in the inner loop, the selected optimal hyperparameters then guided the training of the final model on the outer loop’s training set, and performance was evaluated by pooling outer test set predictions. Classification performance was assessed through sensitivity, specificity, overall accuracy, and the area under the receiver operating characteristic curve (AUC). We also constructed step-wise ROC curves. To determine the statistical significance of the pooled AUC, we performed a nonparametric permutation test with 5,000 iterations, setting the significance level at *P* < 0.05. Specific parameters details are provided in Supplementary method.

## 3 Results

### 3.1 Demographic and clinical characteristics

Participant demographics and clinical characteristics for the three groups (CN, EMCI, LMCI) are presented in Table 1. The groups were well-matched for age (*P* = 0.484), sex (*P* = 0.235), and years of education (*P* = 0.610). In contrast, and as expected, significant group differences emerged for all primary cognitive and biomarker variables, including MMSE, MoCA, ADAS13, APOE ε4 status, Aβ42, and p-tau (all *P* < 0.001). Detailed statistics from these comparisons, including all *post-hoc* results, are provided in Supplementary Table 1.

**TABLE 1:** Demographic and clinical characteristics of CN, EMCI, LMCI groups.

Variable	CN (*N* = 67)	EMCI (*N* = 83)	LMCI (*N* = 58)	*P*-value
Age	71.48 ± 5.93	72.78 ± 6.78	72.00 ± 7.20	0.484^a^
Gender (Male)	34 (50.7%)	51 (61.4%)	28 (48.3%)	0.235^c^
MMSE	30.00 (29.00, 30.00)	29.00 (28.00, 30.00)	28.00 (26.00, 29.00)	**< 0.001^b^**
MoCA	26.28 ± 2.56	23.69 ± 3.22	21.64 ± 3.16	**< 0.001^b^**
ADAS13	8.62 ± 4.51	13.56 ± 5.06	20.06 ± 6.63	**< 0.001^a^**
APOE E4 Carrier (Carrier)	9 (13.4%)	48 (57.8%)	43 (74.1%)	**< 0.001^c^**
Aβ42	231.97 ± 23.31	143.06 ± 25.27	131.40 ± 23.03	**< 0.001^a^**
EDUCATE	16.76 ± 2.58	16.35 ± 2.76	16.67 ± 2.66	0.610^a^
P-TAU	27.60 (22.38, 38.82)	40.40 (28.82, 59.30)	53.00 (39.62, 71.50)	**< 0.001^a^**

Continuous variables are reported as mean ± standard deviation or median (interquartile range); categorical variables are presented using frequencies (percentages).

^a^ One-way ANOVA;

^b^Kruskal-Wallis H test;

^c^Pearson’s Chi-squared test. Bold values indicate statistically significant results (*p* < 0.05).

### 3.2 Group differences in VBM components

**FIGURE 1:**
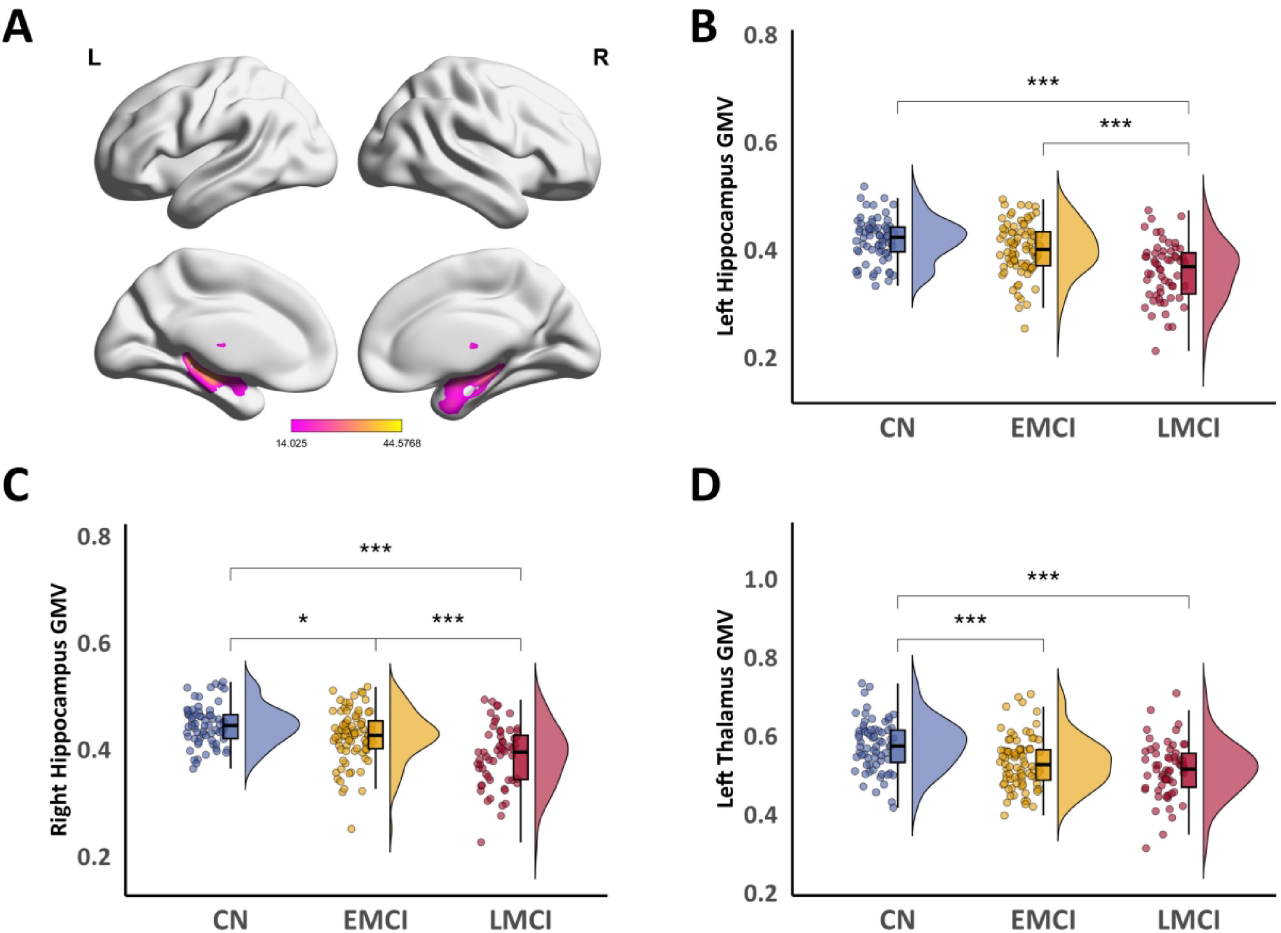
Comparison of GMV differences among CN, EMCI, and LMCI groups. **(A)** Significant GMV clusters identified by ANCOVA among the three groups (Voxel *P* < 0.001, Cluster *P*_FWE_ < 0.05). **(B)** *Post-hoc* comparisons of GMV in the left hippocampal cluster. **(C)** *Post-hoc* comparisons of GMV in the right hippocampal cluster. **(D)** *Post-hoc* comparisons of GMV in the left thalamic cluster. Asterisks denote the level of statistical significance: *P_Bonf_ < 0.05, ***P_Bonf_ < 0.001.

Figure 1A illustrates the significant gray matter volume (GMV) clusters identified by ANCOVA across the three groups. Subsequently, GMV values were extracted from these differential clusters for Bonferroni-corrected *post-hoc* pairwise comparisons. Significantly lower GMV was observed in the left hippocampal cluster (Figure 1B) for the LMCI group compared to both EMCI and CN groups (both *P*_*Bonf*_ < 0.001). No significant difference was found between the CN and EMCI groups (*P*_*Bonf*_ = 0.088). Similarly, in the right hippocampal cluster (Figure 1C), LMCI patients also demonstrated significantly reduced GMV compared to both CN and EMCI groups (both *P*_*Bonf*_ < 0.001). Furthermore, EMCI showed slightly lower GMV than CN (*P*_*Bonf*_ = 0.045). In the left thalamic cluster (Figure 1D), the CN group displayed significantly larger GMV than both EMCI and LMCI groups (both *P*_*Bonf*_ < 0.001), with no significant difference detected between EMCI and LMCI (*P*_*Bonf*_ = 0.564) (Detailed reports are provided in Supplementary Table 2).

### 3.3 Group differences in surface-based morphometry components

**FIGURE 2:**
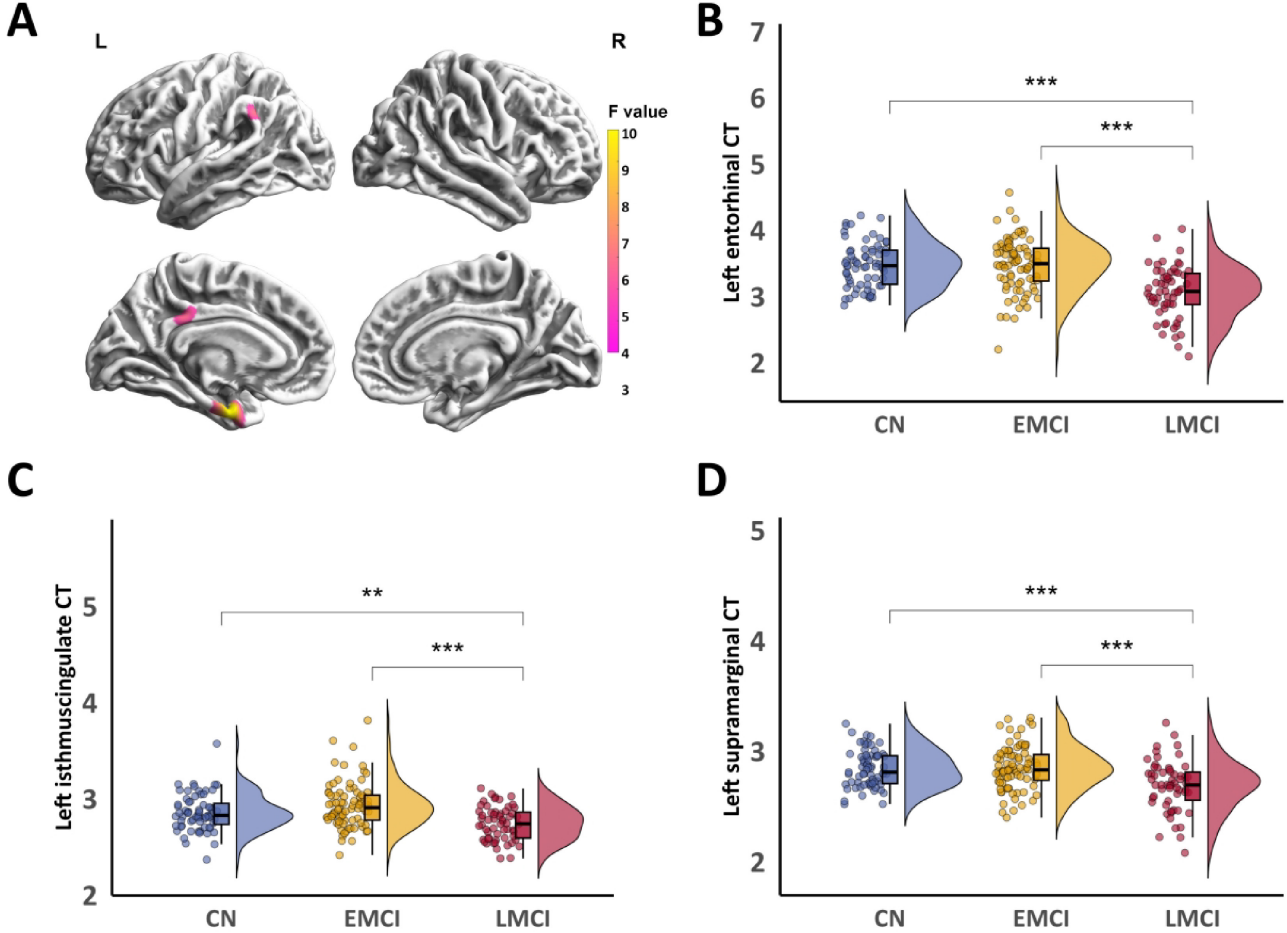
CT differences among CN, EMCI, and LMCI groups. Significant clusters of CT differences identified by ANCOVA among the three groups. **(B)** *Post-hoc* comparisons of CT in the left entorhinal cluster. **(C)** *Post-hoc* comparisons of CT in the left isthmus cingulate cluster. **(D)** *Post-hoc* comparisons of CT in the left supramarginal cluster. Asterisks denote the level of statistical significance: **P_Bonf_ < 0.01, ***P_Bonf_ < 0.001.

Figure 2A illustrates three significant clusters of cortical thickness (CT) differences among the CN, EMCI, and LMCI groups, all located in the left hemisphere. In the left entorhinal cluster (Figure 2B), LMCI showed significantly reduced cortical thickness compared to both CN and EMCI groups (both *P*_*Bonf*_ < 0.001). No significant difference was found between CN and EMCI. For the left isthmus cingulate cluster (Figure 2C), the CN and EMCI groups were comparable (*P*_*Bonf*_ = 0.12), and both exhibited significantly greater cortical thickness than the LMCI group (*P*_*Bonf*_ = 0.006 and *P*_*Bonf*_ < 0.001). In the left supramarginal cluster (Figure 2D), CN and EMCI were again similar (*P*_*Bonf*_ > 0.999), with both groups showing significantly thickness than the LMCI group (both *P*_*Bonf*_ < 0.001). No other significant differences were found for other SBM metrics. All reported *P*-values are Bonferroni corrected. (Detailed reports are provided in Supplementary Table 3).

### 3.4 KLS network

#### 3.4.1 global graph metrics

**FIGURE 3:**
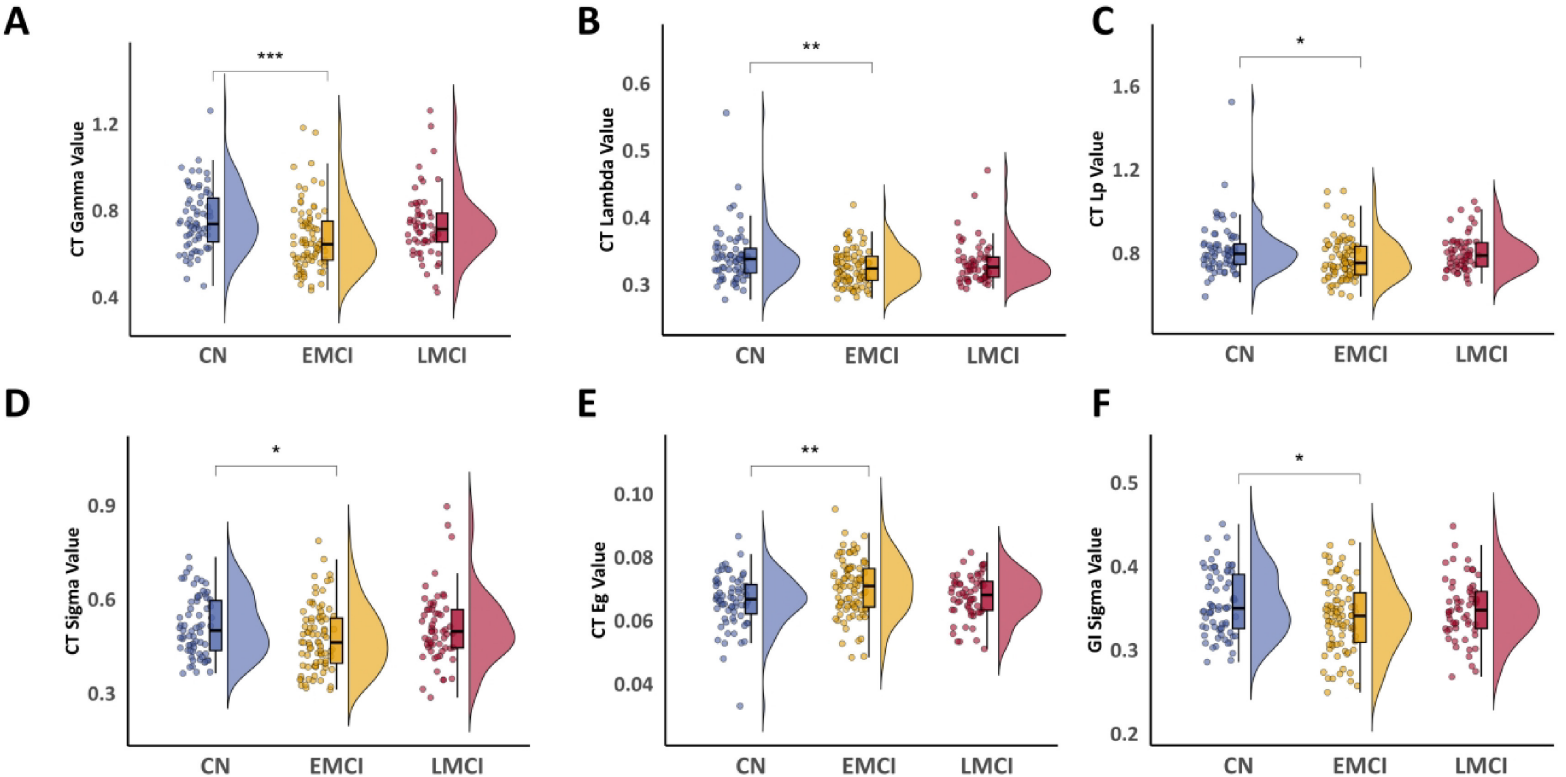
Comparison of global network properties among CN, EMCI, and LMCI groups. **(A)** *Post-hoc* comparisons of Gamma in CT. **(B)** *Post-hoc* comparisons of Lambda in CT. **(C)** *Post-hoc* comparisons of Lp value in CT. **(D)** *Post-hoc* comparisons of Sigma in CT. **(E)** *Post-hoc* comparisons of Eg in CT. **(F)** *Post-hoc* comparisons of Sigma value in GI. Statistical significance: *P_Bonf_ < 0.05, **P_Bonf_ < 0.01, ***P_Bonf_ < 0.001.

Group comparisons of global network metrics derived from the KLS network, constructed using GMV and four SBM indicators, revealed several significant differences. For metrics derived from cortical thickness (CT), the CN group showed significantly higher gamma (*P*_*Bonf*_ < 0.001), lambda (*P*_*Bonf*_ < 0.01), local efficiency (Lp) (*P*_*Bonf*_ < 0.05), and sigma (*P*_*Bonf*_ < 0.05) compared to the EMCI group (Figures 3A–D). We observed that global efficiency (Eg) was significantly elevated in the EMCI group compared with CN (*P*_*Bonf*_ < 0.01) (Figure 3E). For metrics based on the local gyrification index (GI), the CN group exhibited greater small-worldness (sigma value) than EMCI (*P*_*Bonf*_ < 0.05) (Figure 3F). No other global metrics yielded significant differences when comparing EMCI with LMCI, or CN with LMCI (all *P*_*Bonf*_ > 0.05). Bonferroni correction was applied to all *P*-values. Network density analysis with FDR-corrected pairwise comparisons revealed no systematic differences between EMCI and LMCI (significant at only 1 of 26 thresholds, with LMCI showing higher density at this threshold; Supplementary Table 4 and Supplementary Figure 1).

#### 3.4.2 Local topological characteristics

**FIGURE 4:**
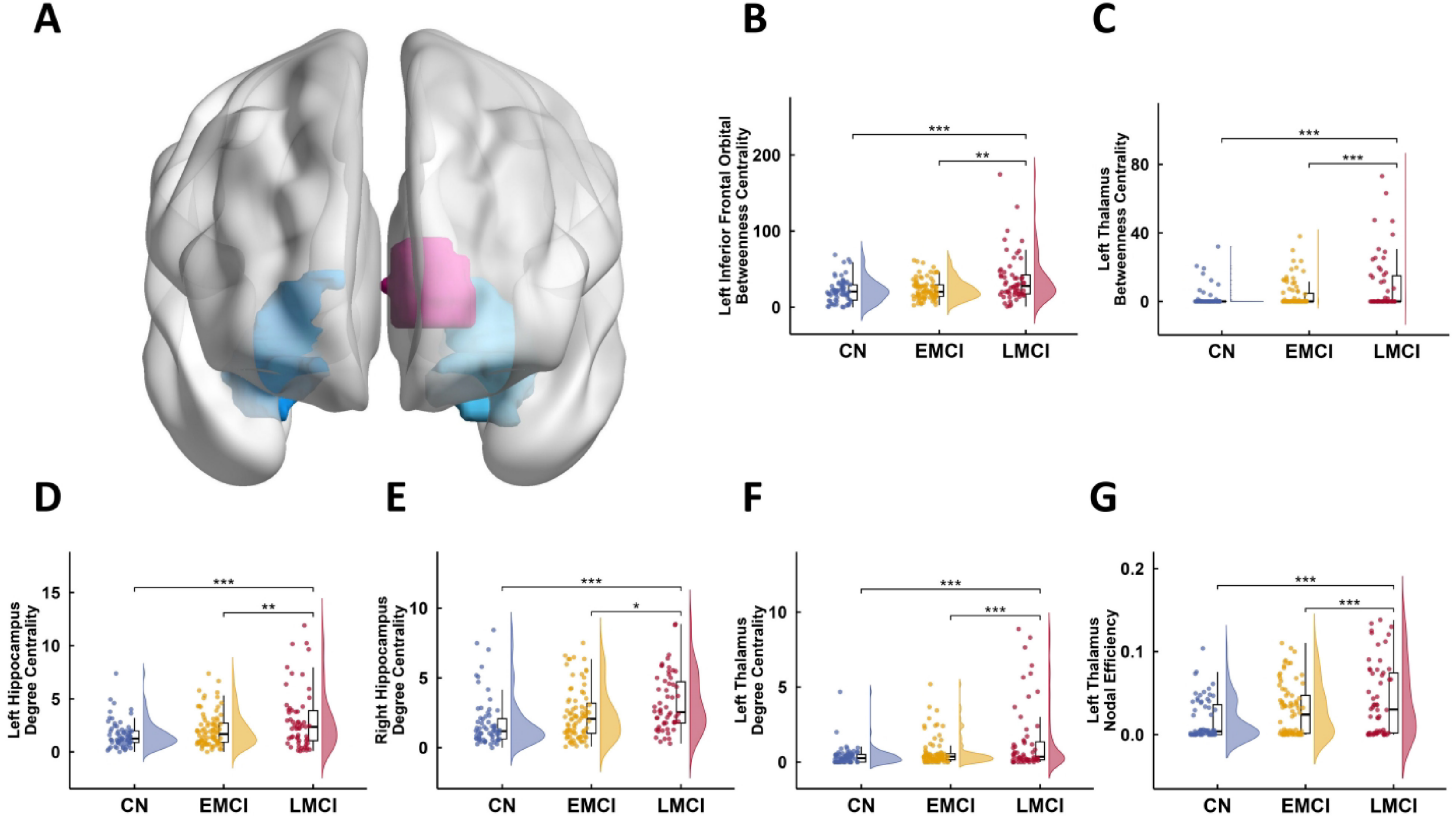
Comparison of local network properties based on GMV among CN, EMCI, and LMCI groups. **(A)** Regions with Significant Nodal Metric Differences. **(B)** *Post-hoc* comparisons of betweenness centrality (BC) in the left inferior frontal orbital gyrus. **(C)** *Post-hoc* comparisons of BC in the left thalamus. **(D)** *Post-hoc* comparisons of degree centrality (DC) in the left hippocampus. **(E)** *Post-hoc* comparisons of DC in the right hippocampus. **(F)** *Post-hoc* comparisons of DC in the left thalamus. **(G)** *Post-hoc* comparisons of nodal local efficiency (NLE) in the left thalamus. Asterisks denote the level of statistical significance: *P_Bonf_ < 0.05, **P_Bonf_ < 0.01, ***P_Bonf_ < 0.001.

Figure 4A illustrates significant group differences in several nodal graph theory metrics based on GMV (*P*_*FDR*_ < 0.05). For betweenness centrality (BC), both CN and EMCI groups showed significantly lower values than the LMCI group in the left inferior frontal orbital gyrus (*P*_*Bonf*_ < 0.001 and *P*_*Bonf*_ < 0.01, respectively) and the left thalamus (both *P*_*Bonf*_ < 0.001). No significant difference was found between CN and EMCI in either region (Figures 4B,C). For degree centrality (DC), both CN and EMCI again exhibited significantly lower values than the LMCI group in the left hippocampus (*P*_*Bonf*_ < 0.001 and *P*_*Bonf*_ < 0.01, respectively), right hippocampus (*P*_*Bonf*_ < 0.001 and *P*_*Bonf*_ < 0.05, respectively), and left thalamus (both *P*_*Bonf*_ < 0.001). No significant difference was observed between CN and EMCI in any of these regions (Figures 4D–F). For nodal local efficiency (NLE) in the left thalamus, both CN and EMCI groups demonstrated significantly lower values compared to the LMCI group (both *P*_*Bonf*_ < 0.001), with no significant difference between CN and EMCI (Figure 4G). All reported *P*-values are Bonferroni corrected.

#### 3.4.3 Network connectivity analysis

**FIGURE 5:**
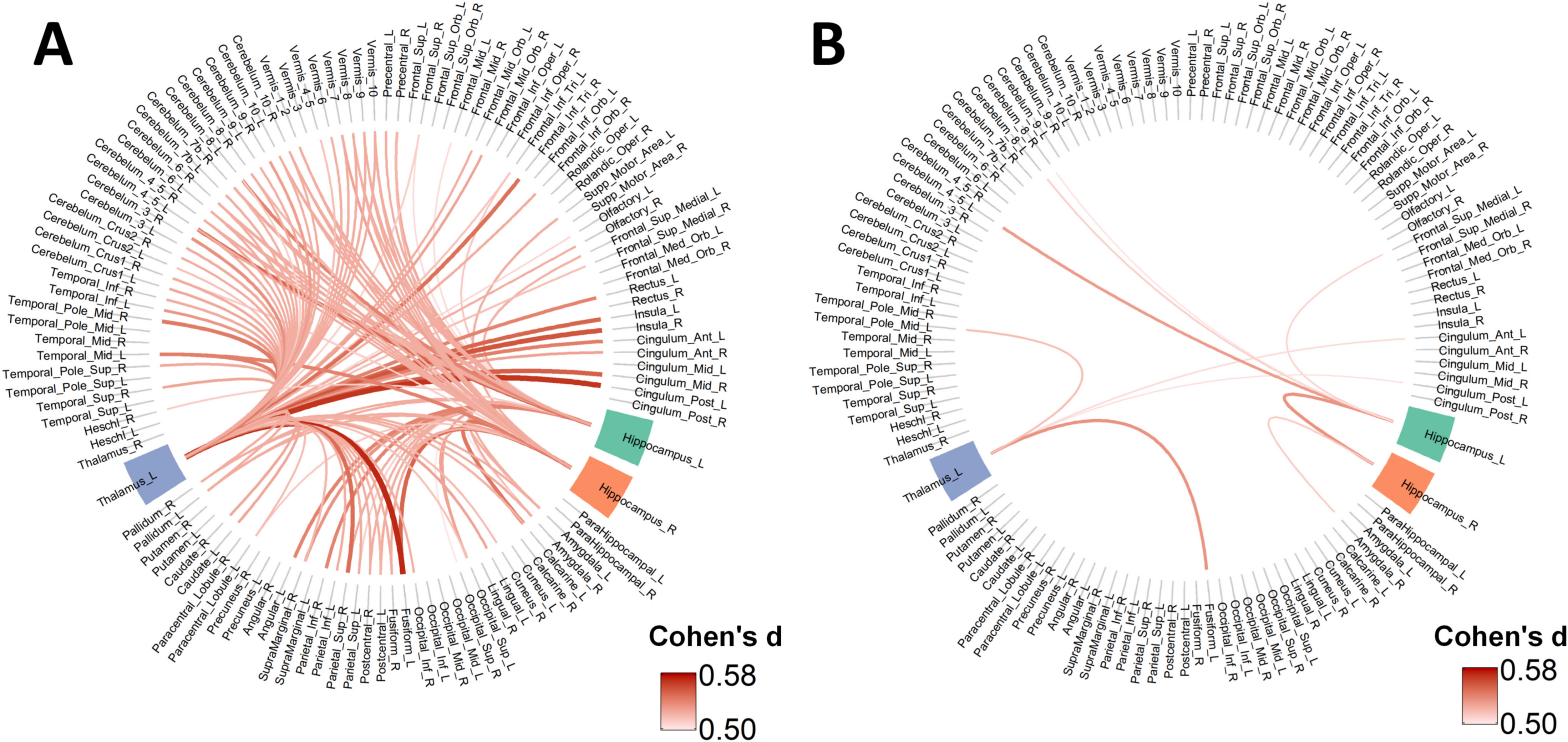
Group differences in structural covariance connections originating from key ROIs. **(A)** Connections with significantly stronger covariance in the CN group compared to the EMCI group. **(B)** Connections with significantly stronger covariance in the CN group compared to the LMCI group. The chord diagrams display structural covariance connections that showed significant group differences (*P*_FDR_ < 0.05) with a large effect size (Cohen’s d > 0.5) in pairwise comparisons.

Our ANCOVA analysis identified significant group differences across many of the 6,670 functional connections (*P*_*FDR*_ < 0.05). *Post-hoc* tests showed that the CN group differed significantly from both the EMCI (144 connections) and LMCI (92 connections) groups. In contrast, we found almost no difference between the EMCI and LMCI groups themselves (only 3 connections). For all significant findings, connectivity was always stronger in the CN group. For visualization, connections with a large effect size (Cohen’s *d* > 0.5) are displayed. Figure 5A illustrates the connections where the CN group showed significantly greater strength than the EMCI group. Similarly, Figure 5B displays the connections where the CN group had significantly greater strength than the LMCI group. The complete list of all significantly altered connections is provided in Supplementary Table 5.

### 3.5 Correlation analysis

**FIGURE 6:**
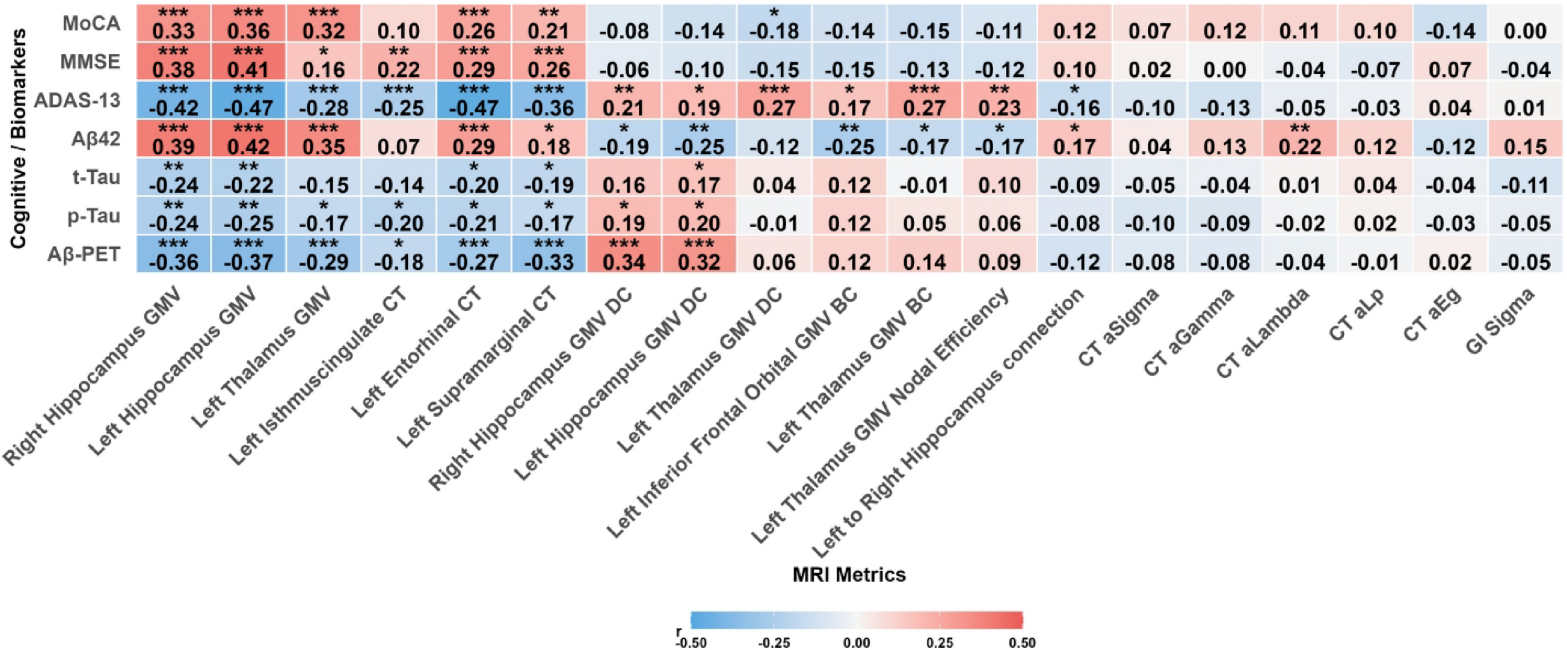
Heatmap of correlations between multimodal imaging metrics and cognitive scores. Statistical significance: *P_FDR_ < 0.05, **P_FDR_ < 0.01, ***P_>FDR_ < 0.001.

Correlation analyses revealed a clear dissociation between the clinical relevance of structural metrics and nodal network properties (Figure 6). Structural integrity metrics, including GMV and cortical thickness in all significant regions, demonstrated a robust association with better cognitive function. Specifically, these metrics were positively correlated with MoCA and MMSE scores (r ranging from 0.16 to 0.41, all *P*_*FDR*_ < 0.05) and negatively correlated with ADAS-Cog-13 scores (r ranging from −0.25 to −0.47, all *P*_*FDR*_ < 0.001). In stark contrast, nodal centrality metrics (BC and DC) and nodal efficiency from the GMV-based network exhibited an opposite relationship. Higher centrality and efficiency were associated with poorer cognitive performance, reflected in a negative correlation with MoCA (*r* = −0.18, *P*_*FDR*_ < 0.05) and positive correlations with ADAS-Cog-13 (r ranging from 0.17 to 0.27, all *P*_*FDR*_ < 0.05). Among inter-regional connections, only the structural covariance between the left and right hippocampus was significantly associated with cognition, showing a negative correlation with ADAS-Cog-13 (*r* = −0.16, *P*_*FDR*_ < 0.05). Finally, no significant correlations were found between any of the global network metrics and cognitive performance. Partial correlation analyses with AD fluid and imaging biomarkers (CSF Aβ42, t-Tau, p-Tau, and amyloid PET SUVR) revealed a similar dissociation: structural metrics were positively correlated with Aβ42 and negatively correlated with tau and amyloid PET, whereas nodal centrality metrics showed the opposite direction of associations (Figure 6 and Supplementary Table 6).

### 3.6 Classification performance of combined imaging markers

**FIGURE 7:**
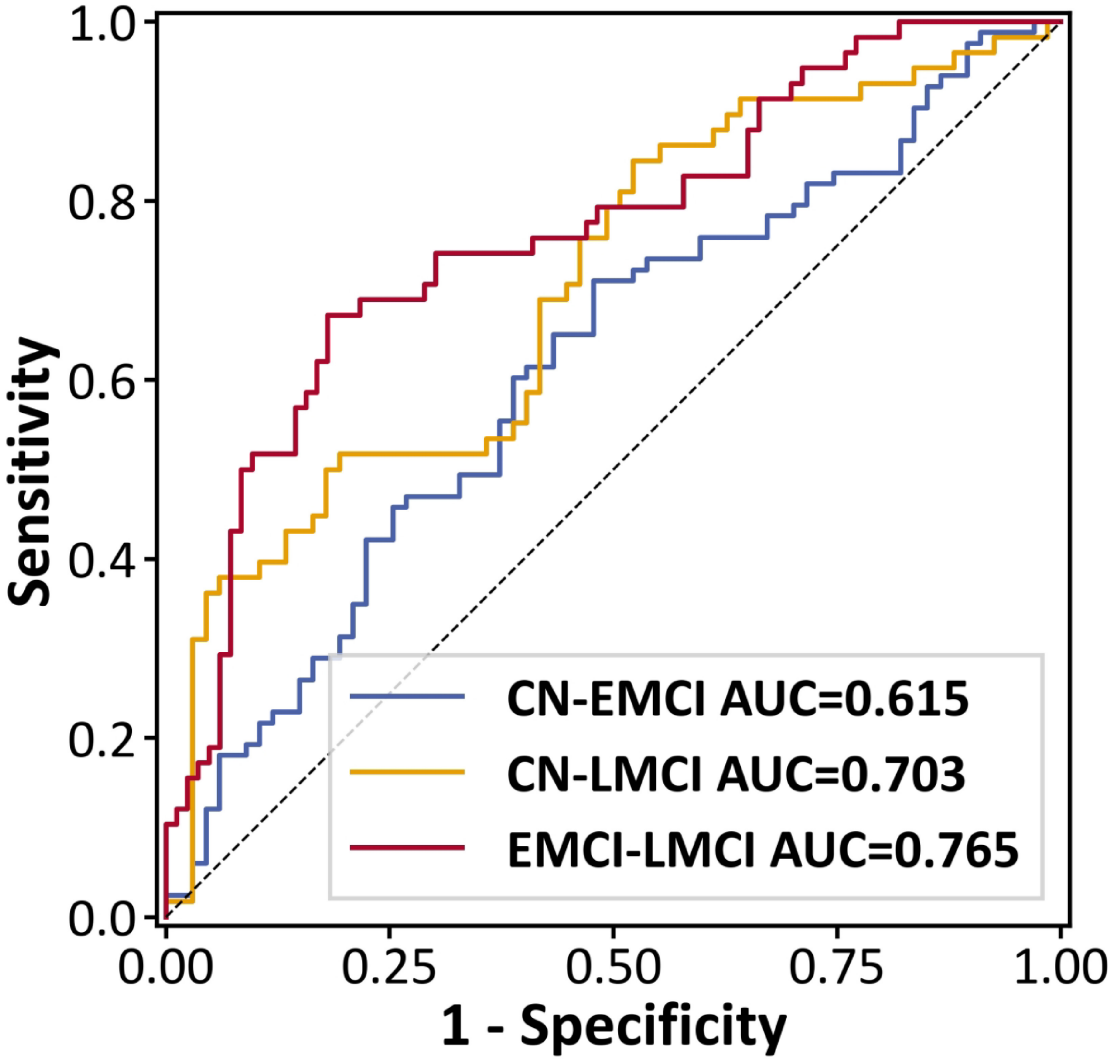
ROC curves for pairwise group comparisons.

All pairwise comparisons showed statistically significant classification performance by permutation testing. The EMCI–LMCI model had the highest area under the ROC curve (AUC = 0.765, permutation test *P* < 0.001). CN–LMCI achieved an AUC of 0.703 (*P* < 0.001). CN–EMCI was lower but remained significant (AUC = 0.615, *P* = 0.021). At the Youden-optimal thresholds, the EMCI–LMCI classifier reached 75.9% accuracy (sensitivity 67.2%, specificity 81.9%), and the CN–LMCI classifier reached 67.2% accuracy (sensitivity 51.7%, specificity 80.6%). Summary metrics are reported in Table 2, and ROC curves are shown in Figure 7. Our analysis of feature selection frequency revealed that the model’s performance was driven by a mix of local morphometric and nodal network markers. The full list of selected features and their frequencies can be found in Supplementary Table 7.

**TABLE 2:** Classification performance of the machine learning model.

Task	AUC^1^	Accuracy	Sensitivity	Specificity	*P*-value
CN—EMCI	0.615	0.627	0.711	0.522	0.021
CN—LMCI	0.703	0.672	0.517	0.806	< 0.001
EMCI—LMCI	0.765	0.759	0.672	0.819	< 0.001

^1^AUC indicates the Area Under the Curve and its 95% Confidence Interval.

## 4 Discussion

In this study, we used a multi-metric analytic framework that combined structural MRI morphometric measures with individual-level structural covariance network (SCN) metrics to characterize differences across MCI subtypes. Morphometric indices showed a monotonic CN–EMCI–LMCI gradient of atrophy. We find a pattern suggestive of a two-stage process in MCI progression. Specifically, patients with EMCI displayed a trend toward increased randomness in their global network architecture. This likely corresponds to an initial, diffuse breakdown in structural connectivity. Conversely, the LMCI stage was characterized by a rise in local network metrics, signaling a shift toward more focal atrophy and subsequent reorganization of the network. The correlation analyses and machine-learning classification further underscore the clinical relevance of these distinct morphometric and network-based markers for differentiating MCI subtypes.

Right hippocampal volume decreased progressively from CN to EMCI to LMCI, whereas left hippocampal volume loss was apparent only in LMCI. This pattern is consistent with the known early vulnerability of the hippocampus to neurofibrillary pathology, which makes hippocampal volume a sensitive marker even at early disease stages (Frisoni et al., 2010; Chen et al., 2022). We also found thalamic atrophy in both MCI groups. The thalamus is often studied in the context of affective or seizure disorders, but it also supports hippocampo-cortical memory circuits (Strange et al., 2014; Aggleton et al., 2016). Its atrophy here may contribute to memory deficits by disrupting this subcortical relay (Cassel et al., 2024).

SBM analysis showed cortical thinning primarily in LMCI, localized to three left-hemisphere regions: entorhinal cortex, isthmus cingulate, and supramarginal gyrus. The entorhinal cortex is a gateway to the hippocampal memory system and accumulates tau pathology early (Moscovitch et al., 2016; Mieling et al., 2025). The isthmus cingulate, a posterior hub of the default mode network, is central to episodic memory (Cieri et al., 2021; Cieri et al., 2022). The supramarginal gyrus supports complex cognition (Chen Z. et al., 2024). Both atrophy and thinning occur in AD, and we saw early atrophy signs in EMCI, though which comes first remains unclear (Li et al., 2025).

Compared to controls, the EMCI group had disrupted small-world properties and a more randomized network configuration—a pattern seen across several neuropsychiatric conditions (Bullmore and Sporns, 2012; Liu et al., 2013; Suo et al., 2018; Frantzidis et al., 2014). Surprisingly, these global differences were less pronounced in LMCI, which looked similar to both CN and EMCI on these measures. Local metrics, however, showed a different picture: nodal centrality (BC and DC) was elevated in the hippocampus, thalamus, and orbital inferior frontal gyrus—exclusively in LMCI. Increased nodal importance in degenerating regions may seem paradoxical, but it has been reported in other disorders as a sign of network dysfunction (Chen W. et al., 2024; Zhang et al., 2023). Elevated DC and BC in regions such as the hippocampus, coupled with the reduced group differences in global properties, may not be viewed as a sign of recovery in LMCI group, but rather as a marker of a structurally and functionally compromised network. We observed significant global network differences primarily in cortical thickness and gyrification, not across all metrics. This heterogeneity supports, rather than undermines, our multi-metric framework. Given that cortical thinning and gyrification likely follow different pathological timelines than volumetric atrophy (Li et al., 2025), a single-metric approach would have failed to detect these specific topological disruptions. Thus, these findings underscore the importance of integrating complementary surface-based markers to fully characterize MCI degeneration.

Our analysis also revealed widespread reductions in connectivity in the EMCI group relative to controls. Moreover, mirroring the pattern seen for small-world properties, the differences between LMCI and CN were less pronounced than those between the EMCI and CN at a medium effect size threshold. It is well-established from neuropathological studies that AD pathology spreads in a hierarchical, non-uniform manner: tau pathology typically originates in the medial temporal lobe before spreading to association cortices (Braak and Braak, 1991), while amyloid-β (Aβ) follows a different, though similarly staged, accumulation pattern (Thal et al., 2002). Consistent with these models of staged and imbalanced neurodegeneration, the focal atrophy we observed in the EMCI group may disrupt morphological similarities between brain regions, leading to widespread network decoupling. We speculate that this pattern of more homogenized, widespread atrophy could, paradoxically, increase the relative morphological similarity between different degenerating regions (Risacher et al., 2010; Raj et al., 2012). These observations point to a possible two-stage process along the CN–EMCI–LMCI continuum: early network randomization followed by regional atrophy and reorganization. This sequence fits with theoretical models of AD neurodegeneration, where initial diffuse pathology gives way to more localized network changes (Franzmeier et al., 2020; Jones et al., 2015). Of course, our cross-sectional design limits any causal interpretation. Longitudinal imaging will be essential to test this hypothesis.

We analyzed the relationship between the imaging metrics and cognitive function. GMV and cortical thickness correlated with better cognitive performance. Higher nodal centrality in the hippocampus and thalamus, by contrast, was linked to worse cognitive scores. The biomarker analysis supports that patients with elevated degree centrality (DC) also tended to have lower CSF Aβ42 and higher levels of p-tau, t-tau, and amyloid PET SUVR. In other words, increased centrality tracks with increased pathology. In short, increased centrality tracked with increased pathology. This makes it unlikely that elevated centrality in LMCI represents compensation; it appears to be a maladaptive response (Seeley et al., 2009). Similar patterns of pathological network reorganization have been reported in fMRI studies (Tijms et al., 2011).

To evaluate whether a combination of structural and network features could improve MCI subtype classification, we trained a logistic regression model. The model achieved an AUC of 0.765 for EMCI vs. LMCI, supporting the value of a multi-metric approach for patient stratification (Petersen, 2004; Wei et al., 2025; Veitch et al., 2018). The model separated EMCI from LMCI more accurately than it separated CN from either MCI group. The transition from EMCI to LMCI may involve sharper network and atrophy shifts, while the CN–MCI boundary is more gradual or variable (Iturria-Medina et al., 2016; Jack et al., 2013). This fits with prior reports of non-linear network changes across the AD spectrum (Jones et al., 2015). The lower CN vs. LMCI accuracy could also stem from overlapping features or heterogeneity within those groups. Larger longitudinal cohorts will be needed to clarify these dynamics and validate our findings.

### 4.1 Limitations

Several limitations should be acknowledged. First, as a cross-sectional investigation, our study precludes causal inference. Second, the moderate size and single-center origin of our sample constrain the generalizability of our findings, implying that the machine learning results are best considered exploratory. Finally, our network neuroscience approach involved specific methodological choices. The node definition depended on the chosen parcellation atlas, and network properties can vary across different schemes. While we selected the KLS framework based on its established validation and reliability, alternative approaches such as Jensen-Shannon divergence offer comparable properties (e.g., inherent symmetry and boundedness). A systematic comparison of different similarity metrics for individual morphological networks would be a valuable direction for future research.

## 5 Conclusion

In conclusion, Our study identifies a distinct two-stage MCI trajectory: initial global network randomization in EMCI, followed by focal atrophy and nodal reorganization in LMCI. These structural shifts track closely with cognitive decline and AD pathology. By combining these complementary features, we successfully distinguished MCI subtypes, underscoring the value of multi-level profiling for refined patient stratification in clinical trials.

## Data Availability

The original contributions presented in this study are included in the article/Supplementary material, further inquiries can be directed to the corresponding author.

## Footnotes

1
http://www.neuro.uni-jena.de/cat12

2
https://www.mathworks.com/matlabcentral/fileexchange/14034-kernel-density-estimator

3
https://www.nitrc.org/projects/gretna/
