# Correction: Distinguishing early from late mild cognitive impairment: a multi-level analysis of regional morphometry and KLS-derived network topology

**DOI:** 10.3389/fnagi.2026.1828194

**Published:** 2026-04-01

**Authors:** Peng Yan, Xinyu Du, Siyu Yang

**Affiliations:** 1 Department of Neurology, The First Affiliated Hospital with Nanjing Medical University, Nanjing, Jiangsu, China; 2 Department of Neurology, The Second Hospital of Nanjing, Affiliated to Nanjing University of Chinese Medicine, Nanjing, Jiangsu, China

**Keywords:** individual structural covariance network, Kullback-Leibler similarity, machine learning, mild cognitive impairment, structural magnetic resonance imaging

In the published article, affiliation 2 was erroneously given as: “The Second Hospital of Nanjing, Affiliated to Nanjing University of Chinese Medicine, Nanjing, Jiangsu, China.” The corrected affiliation 2 should have been: “Department of Neurology, The Second Hospital of Nanjing, Affiliated to Nanjing University of Chinese Medicine, Nanjing, Jiangsu, China.”

The original version of this article has been updated.

