# Supplementary material for: Distinguishing early from late mild cognitive impairment: a multi-level analysis of regional morphometry and KLS-derived network topology
Source: Front Aging Neurosci. 2026 Mar 11;18:1730305. doi: 10.3389/fnagi.2026.1730305 (PMC13012939; doi:10.3389/fnagi.2026.1730305)
Supplement: Supplementary file 1 [file Data_Sheet_1.pdf]

Supplementary Material

1 Supplementary Methods :

Machine learning analyses： To further validate the observed between-group differences in imaging features and to investigate their potential diagnostic utility, Logistic Regression (LR) classifiers were employed to construct models for distinguishing pairwise subject groups (CN vs. EMCI, CN vs. LMCI, EMCI vs. LMCI). Covariate Control via Per-fold Residualization: To rigorously control for potential confounding factors, a "Per-fold Residualization" strategy was implemented within the machine learning pipeline. In each fold of the outer cross-validation, separate linear regression models were fitted solely on the training set data. For each imaging feature, a linear model was constructed against specified covariates, which included Sex, APOE Risk, TIV, Age, and educate year(1, 2). Categorical variables (Sex, APOE Risk) were dummy-encoded for this process. Notably, for SBM-related features such as CT aSigma and CT aEg, TIV_Z was deliberately excluded from the covariate model during residualization to avoid over-correction. Subsequently, the residuals, obtained by subtracting the linear model's predictions from the original feature values, served as the new, decorrelated features for both the training and test sets within that specific fold. This per-fold residualization approach was critical in preventing information leakage from the test set(3).

Nested Cross-Validation and Hyperparameter Optimization: The machine learning process employed a robust nested cross-validation (CV) scheme to ensure reliable performance estimation and unbiased hyperparameter tuning(4). The outer loop consisted of 10-fold stratified CV, dedicated to evaluating the model's generalization performance. Within each outer training fold, an inner loop of 5-fold stratified CV was used for optimizing the LR model's hyperparameters. Specifically, within each outer training fold, an initial feature selection step was performed using L1-regularized Logistic Regression (LASSO), where only features with non-zero coefficients from the best-performing LASSO model (optimized in the inner loop) were retained for the subsequent training of the final L2-regularized LR classifier.

Model Training, Validation, and Statistical Significance: Upon the completion of hyperparameter tuning within the inner loop, the identified optimal hyperparameters were then used to train the final LR model. This training was performed on the full training set of the outer loop, utilizing the residualized and normalized data. Model validation was subsequently conducted on the independent testing set of the outer loop. Classification performance was comprehensively assessed using standard metrics, including total accuracy, AUC, sensitivity, and specificity. Receiver Operating Characteristic (ROC) curve analysis was systematically performed, with step-wise ROC curves constructed to visualize classifier performance across various thresholds. To estimate the statistical significance of the pooled AUC, nonparametric permutation tests were conducted with 5,000 permutations, with statistical significance set at $p < 0.05$(5). All analyses were implemented in Python, primarily leveraging the scikit-learn and statsmodels libraries. A fixed random seed (42) was consistently used throughout the analyses to ensure full reproducibility.

Reference：

1. Fortin Jean-Philippe, Cullen Nicholas, Sheline Yvette I., Taylor Warren D., Aselcioglu Irem, Cook Philip A., et al. Harmonization of cortical thickness measurements across scanners and sites [J]. Neuroimage, 2017, 167: 104-120. doi: 10.1016/j.neuroimage.2017.11.024
2. Cole James H., Poudel Rudra P. K., Tsagkrasoulis Dimosthenis, Caan Matthan W. A., Steves Claire, Spector Tim D., et al. Predicting brain age with deep learning from raw imaging data results in a reliable and heritable biomarker [J]. Neuroimage, 2017, 163: 115-124. doi: 10.1016/j.neuroimage.2017.07.059
3. Bernett Judith, Blumenthal David B., Grimm Dominik G., Haselbeck Florian, Joeres Roman, Kalinina Olga V., et al. Guiding questions to avoid data leakage in biological machine learning applications [J]. Nat Methods, 2024, 21(8): 1444-1453. doi: 10.1038/s41592-024-02362-y
4. Varma Sudhir, Simon Richard. Bias in error estimation when using cross-validation for model selection [J]. BMC Bioinf, 2006, 7: 91. doi:
5. Ojala Markus, Garriga Gemma C. %J IEEE. Permutation Tests for Studying Classifier Performance [J]. 2009. doi:

2 Supplementary Figure :

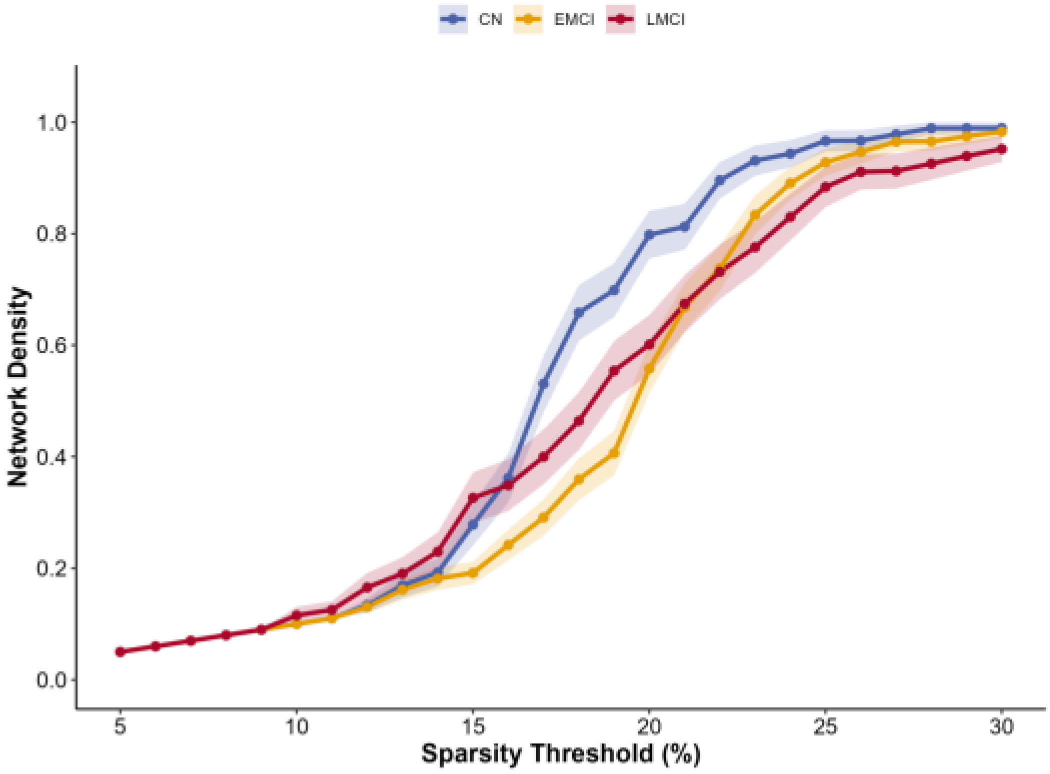

Supplementary Figure S1. Network density-sparsity curves across groups.
